# Surgical site infection in paediatric posterior fossa surgery: does pathology matter?

**DOI:** 10.1007/s00381-021-05131-w

**Published:** 2021-04-10

**Authors:** Malik Zaben, Alexandra Richards, Joseph Merola, Chirag Patel, Paul Leach

**Affiliations:** 1 Department of Neurosurgery, University Hospital of Wales, Cardiff, UK; 2 Neuroscience and Mental Health Research Institute, School of Medicine, Cardiff University, Institute of Psychological Medicine and Clinical Neurosciences, Room 4FT 80E, 4th Floor, University Hospital Wales, Heath Park, Cardiff, CF14 4XN UK

**Keywords:** Surgical site infection, Medulloblastomas, Posterior fossa

## Abstract

**Objectives:**

The aim of this study was to explore the rates and potential risks of surgical site infection (SSI) after posterior fossa surgery for tumour resection in children.

**Methods:**

We retrospectively reviewed our local paediatric (age < 16 years) database for all cases of posterior fossa (PF) brain tumour surgery between November 2008 and November 2019. We collected patient demographics, tumour histology/location, and the event of postoperative surgical site infection.

**Results:**

Overall, 22.1% (*n*=15) developed SSI out of sixty-eight children undergoing PF surgery for resection of brain tumours; 73.3% of them had a confirmed diagnosis of medulloblastoma. There was no statistically significant difference in the age (5.1 ± 0.60 vs. 6.2 ± 0.97 years; *p*=0.47) and duration of operation (262 vs. 253 min; *p* = 0.7655) between the medulloblastoma group and other tumours. Although the rate of postoperative hydrocephalus was higher in the medulloblastoma group (12.9% vs. 0%), this was not associated with increased SSI. Rates of CSF leak between the 2 groups were not different.

**Conclusion:**

Medulloblastoma as a pathological entity seems to carry higher risk of postoperative surgical site infection compared to other types of paediatric posterior fossa tumours. Further larger studies are required to look into this causal relationship and other risk factors that might be involved.

## Introduction

With the exclusion of brainstem tumours, the current standard of care for patients with malignant posterior fossa tumours remains maximal safe surgical resection followed by chemo- and/or radiotherapy [1]. Postoperative surgical site infection (SSI) can however result in a prolonged hospitalisation and potentially a delay in commencement of postop adjuvant therapy. Understanding risk factors for SSI is particularly important given evidence of higher incidence of local infection in PF surgery when compared to its supratentorial counterpart [2]. Very limited literature has however specifically addressed this issue. We, therefore, sought to determine the incidence and potential risk factors for SSI after resection of posterior fossa tumours in children. Our data clearly demonstrates that medulloblastomas carry significantly higher risk of SSI compared with other posterior fossa brain tumours. Such an increased risk is not related to age at the time of surgery, operative time, and/or surgical technique.

## Methods

A retrospective review of the paediatric (age < 16 years) neurosurgical database at our local unit was performed between November 2008 and November 2019 for children that underwent posterior fossa surgery for tumour resection. The following data was extracted: patient demographics, tumour histology/location, and postoperative surgical site infection. General preoperative preparation was consistent with local protocol over the time period of data collection, involving surgical site preparation with alcoholic betadine and administration of antibiotics 30 min prior to skin incision. The standard approach for all cases was midline suboccipital craniotomy, Y-shaped dural opening [1], and watertight dural closure with DuraGen**®** (Integra) and Tisseel fibrin sealant (Baxter). Postoperative infection was defined as the occurrence of wound infection (superficial or deep), meningitis, or ventriculitis during the postoperative period. Statistical analysis was performed using the GraphPad Prism software (Version 8.0.1).

## Results

**Fig. 1:**
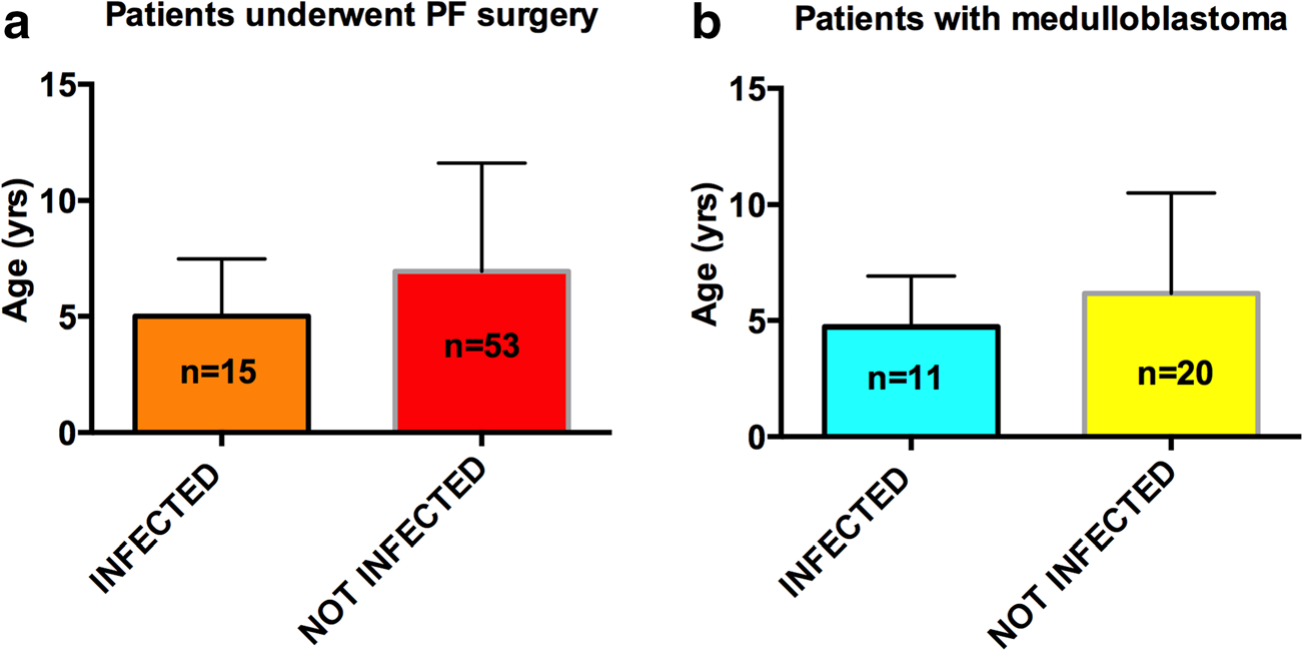
Age as a risk factor of SSI. Age difference between infected and not infected groups **a** in the whole cohort and **b** in the medulloblastoma group. There was no statistical difference in either comparison; *t* student test

**Fig. 2:**
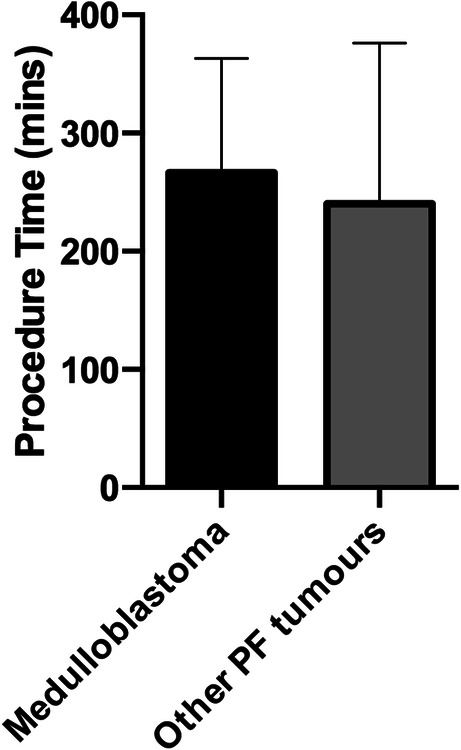
Duration of the surgical operative time in medulloblastoma patients versus the rest of the cohort. There was no statistical difference in either comparison; *t* student test

A total of 68 children underwent PF surgery for resection of brain tumours, with an average age of 7.0 years (SD 4.3 years; range 11 days–15.8 years). Medulloblastoma accounted for the majority of tumours (45.6%), and the remainders are summarised in Table 1. The overall incidence of postoperative infection in the cohort was 22.1% (15 out of 68 children). Of these children, a disproportionately large proportion consisted of patients with medulloblastoma (11 out of 15; 73.3%). Overall, the average age of those who developed infection (5.0 ± 0.6 years, *n*=15) was not statistically different from those who did not (6.943 ± 0.6428, *n*=53; *p*= 0.1277) (Fig. 1a). Similarly, the average age of medulloblastoma patients that developed subsequent infection (5.1 ± 0.60 years) was not statistically different from that in the noninfected subgroup (6.2 ± 0.97 years; *p*=0.47) (Fig. 1b). Only one patient of the medulloblastoma group was complicated with CSF leak; this patient did not develop SSI. Five patients developed hydrocephalus postoperatively (four had medulloblastoma and one ependymoma); none of the medulloblastoma patients developed SSI but the patient with ependymoma did. Only two patients in our cohort developed postoperative CSF leak (one with medulloblastoma and the other with pilocytic astrocytoma); none of them developed SSI. Comparing surgical operative time between medulloblastoma and other posterior fossa tumours revealed no significant differences (262 vs. 253 min; *p* = 0.7655) (Fig. 2). Virtually, all of the patients in the cohort had DuraGen® (Integra Life Sciences) for their duraplasty.

**Table 1:** Summary of paediatric posterior fossa surgery cohort based on final histological diagnosis

Tumour	Number (%)
Medulloblastoma	31 (45.6)
Pilocytic astrocytoma	20 (39.4)
Ependymoma	5 (7.4)
ATRT	4 (5.9)
Pilomyxoid astrocytoma	3 (4.4)
Anaplastic astrocytoma	2 (2.9)
Dermoid cyst	1 (1.5)
Low-grade glioma	1 (1.5)
Diffuse astrocytoma	1 (1.5)

## Discussion

Medulloblastoma is the most common malignant paediatric brain tumour, accounting for 20% of paediatric brain tumours [3]. The standard of care in our unit involves the following: (i) surgical resection of the tumour, aiming for <1.5cm^2^ residual tumour; (ii) tumour staging involving MRI brain and spine, CSF cytology, and molecular subtyping; (iii) chemoradiotherapy in accordance with the outcome of the former two stages. Understanding risk factors for infection following surgery is therefore paramount in order to avoid any unnecessary delays in subsequent chemoradiotherapy. Our findings demonstrate that children with medulloblastoma experience a much higher incidence of SSI. Overall, the rate of SSI in adult cohort studies is higher after posterior fossa surgery (16.2%) compared to supratentorial craniotomies (5–10%). In our paediatric cohort, the risk of infection is around 22%. We examined the most obvious explanation that this may be attributed to prolonged duration of the procedure but found no significant differences between medulloblastoma and other posterior fossa tumours. We also examined whether the higher rate of SSI in the medulloblastoma group was related to the development of postoperative hydrocephalus. Our data suggest that although the medulloblastoma group had a higher rate of infection compared to other pathologies, hydrocephalus was not associated with increased risk of SSI. Likewise, rates of CSF leak were evenly distributed between the two groups; the one patient with CSF leak in the medulloblastoma leak did not develop SSI. Whilst infection could have also been related to the type of synthetic dural substitute used for duraplasty, DuraGen® was used consistently in all our cases and thus unlikely to have contributed to the higher rate of SSI in the medulloblastoma group. The average age of medulloblastoma with a postoperative infection was 5.1 years, compared to 6.2 years in those that did not develop infection (Fig. 1b), indicating that age is not a risk factor for susceptibility to infection in this cohort. On the other hand, this may be related to underlying immunosuppression secondary to medulloblastoma pathogenesis, demonstrated by recent investigation of local tumour immunosuppression [4]. Some studies on the adult cohorts [2] have also identified emergency surgery (as opposed to elective surgery) as a potential risk for SSI; all, but one, of our medulloblastoma cases in this cohort were operated on electively. Whilst this higher risk of infection in the medulloblastoma group could also be related to other factors such as potentially higher dose and longer duration of perioperative steroids in the medulloblastoma group, we have no data to report on this and therefore identify this as a limitation of this study. Larger studies are required to (i) establish whether there children with medulloblastoma are at an increased risk of postoperative infection, (ii) identify risk factors in this cohort, and (iii) optimise pharmacological prophylaxis for treatment (Table 2).

**Table 2:** Cases (*n*=68) grouped based on diagnosis and whether a SSI was recorded or not. A chi-square test was performed to examine the relation between a diagnosis of medulloblastoma vs. other pathological entities and the risk of developing SSI. The relation between these variables was significant, *χ*^2^(1, *N*=68)=5.97, *p*=0.015

Tumour histopathology	Infected cases (*n*) (%)	Not infected cases (*n*) (%)	Total (*n*)
Medulloblastoma	11 (35.5)	20 (64.5)	31
Others	4 (10.8)	33 (89.2)	37
